# Supplementary material for: Humoral and T Cell Immune Responses against SARS-CoV-2 after Primary and Homologous or Heterologous Booster Vaccinations and Breakthrough Infection: A Longitudinal Cohort Study in Malaysia
Source: Viruses. 2023 Mar 25;15(4):844. doi: 10.3390/v15040844 (PMC10146761; doi:10.3390/v15040844)
Supplement: Supplementary file 1 [file viruses-15-00844-s001.zip › Figure S1.pdf]

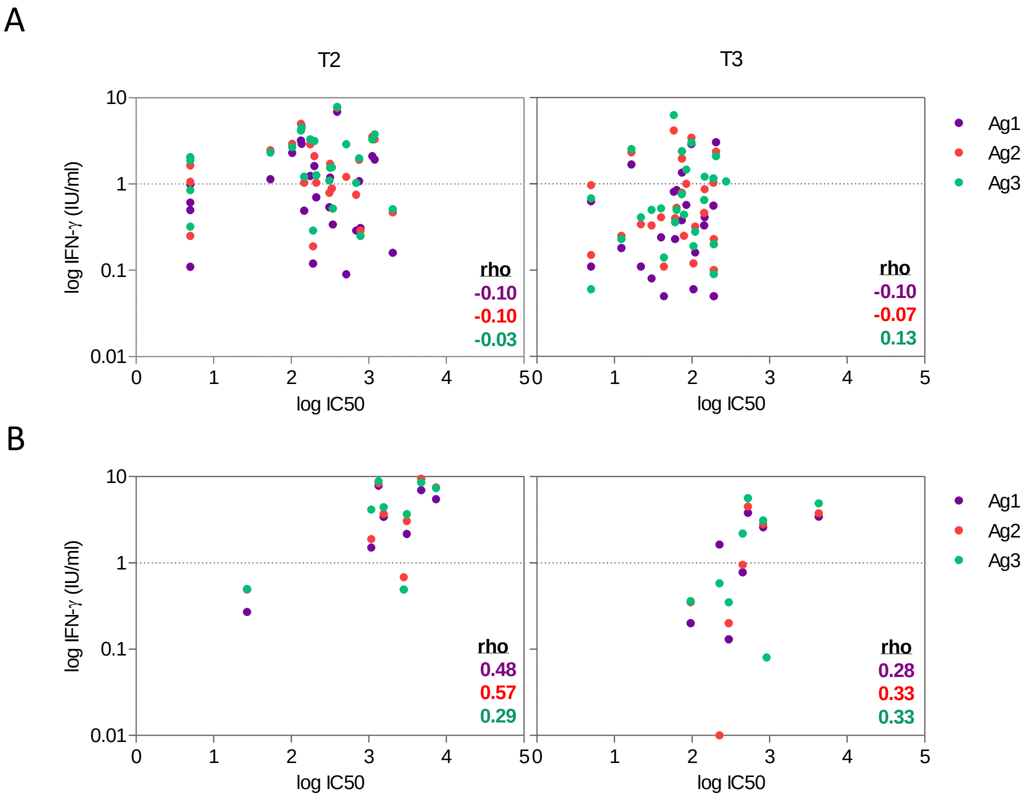

Figure S1. Correlation between neutralizing antibody and T cell responses in (A) vaccinated uninfected (n=25), and (B) post-COVID-19 (n=8) individuals at 21 days (T2) and 3 months (T3) post-second dose. The relationships are presented for Ag1 (purple), Ag2 (red) and Ag3 (green). Correlations were assessed using Spearman's correlation coefficient and expressed as rho.
