# Supplementary material for: Humoral and T Cell Immune Responses against SARS-CoV-2 after Primary and Homologous or Heterologous Booster Vaccinations and Breakthrough Infection: A Longitudinal Cohort Study in Malaysia
Source: Viruses. 2023 Mar 25;15(4):844. doi: 10.3390/v15040844 (PMC10146761; doi:10.3390/v15040844)
Supplement: Supplementary file 1 [file viruses-15-00844-s001.zip › Figure S2.pdf]

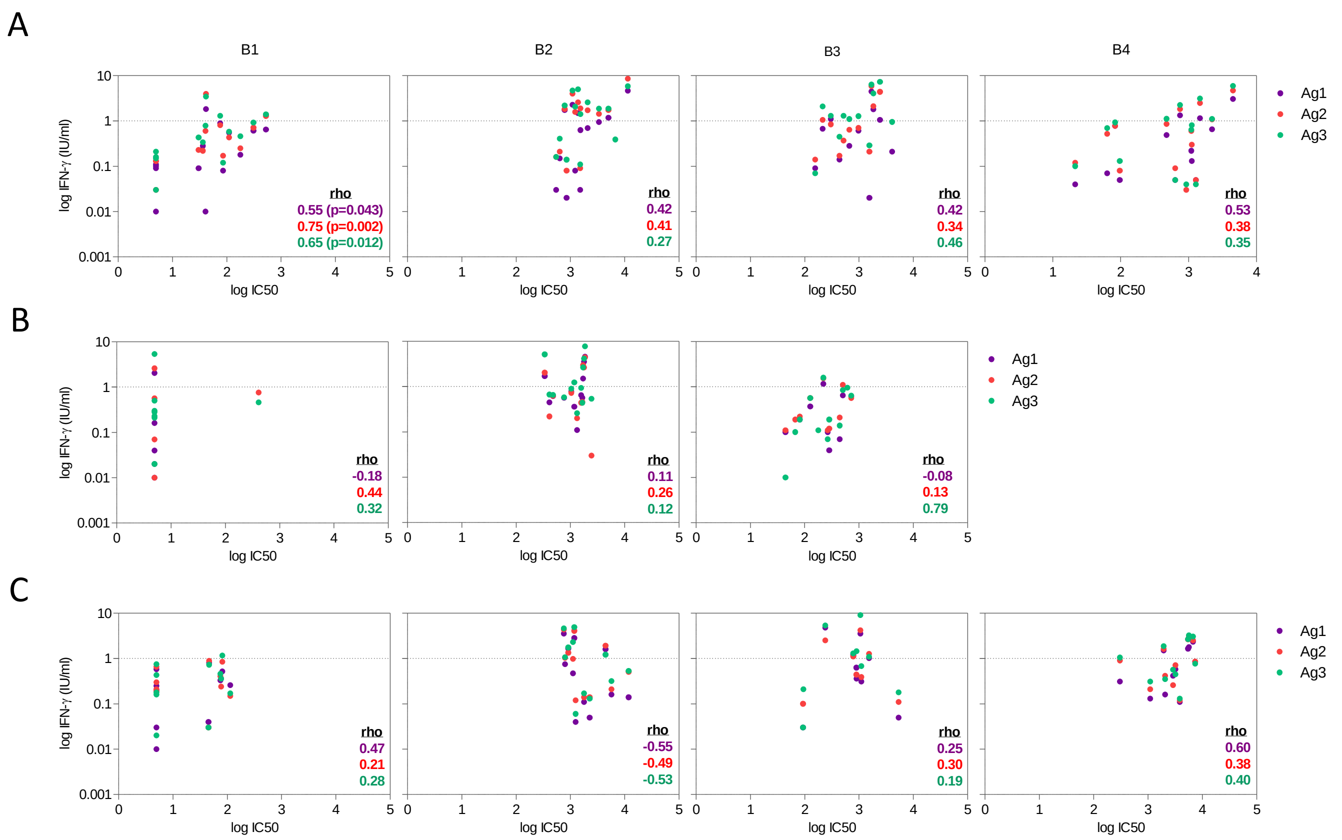

Figure S2. Correlation between neutralizing antibody and T cell responses in individuals receiving (A) homologous (BNT-primed + BNT) (*n*=14) booster, (B) homologous booster with breakthrough infections (*n*=11) and (C) heterologous (ChAd-primed + BNT) (*n*=15) booster at time-points before (B1), 21 days (B2), 3 months (B3) and 6 months (B4) after booster. The relationships are presented for Ag1 (purple), Ag2 (red) and Ag3 (green). Correlation was assessed using Spearman's correlation coefficient and expressed as rho. Significant *p* values for each correlation are denoted.
