# Supplementary material for: Humoral and T Cell Immune Responses against SARS-CoV-2 after Primary and Homologous or Heterologous Booster Vaccinations and Breakthrough Infection: A Longitudinal Cohort Study in Malaysia
Source: Viruses. 2023 Mar 25;15(4):844. doi: 10.3390/v15040844 (PMC10146761; doi:10.3390/v15040844)
Supplement: Supplementary file 1 [file viruses-15-00844-s001.zip › Table S1.pdf]

Table S1. Demographic characteristics of individuals.

Characteristics	Uninfected	Post-COVID-19	BNT162b2 (homologous booster)	ChAdOx1-S - BNT162b2 (heterologous booster)	Breakthrough infection (homologous booster)
Recruited	43	11	30	16	0
Sample size	25	8	14	15	11
Median age, years (IQR)	30 (19-38)	44 (33-60)	38 (35-50)	29 (25-41)	40 (32-46)
Male (%)	14 (56)	4 (50)	3 (21)	4 (27)	1 (9)
**Primary course vaccines, *n* (%)**					
BNT162b2	26 (100)	7 (88)	14 (100)		11 (100)
ChAdOx1-S				15 (100)	
CoronaVac		1 (12)			
**Booster vaccine, *n* (%)**					
BNT162b2			14 (100)	15 (100)	11 (100)
**Comorbidities, *n* (%)**					
Chronic respiratory disease			1 (7)		
Hypertension		3 (38)	1 (7)		4 (36)
Diabetes		1 (13)			
