# Supplementary material for: Humoral and T Cell Immune Responses against SARS-CoV-2 after Primary and Homologous or Heterologous Booster Vaccinations and Breakthrough Infection: A Longitudinal Cohort Study in Malaysia
Source: Viruses. 2023 Mar 25;15(4):844. doi: 10.3390/v15040844 (PMC10146761; doi:10.3390/v15040844)
Supplement: Supplementary file 1 [file viruses-15-00844-s001.zip › Table S2.pdf]

Table S2. Pseudovirus neutralization assay against SARS-CoV-2 wild type (WT) and Omicron spike, Elecsys Anti-SARS-CoV-2 S and Elecsys Anti-SARS-CoV-2 results for vaccinated uninfected individuals and post-COVID-19 patients before (T1), and at 21 days (T2) and 3 months (T3) after the second vaccine dose.

Group	T1				T2				T3			
	WT nAb (IC_{50})	Omicron nAb (IC_{50})	Anti-N (COI)	Anti-S (U/ml)	WT nAb (IC_{50})	Omicron nAb (IC_{50})	Anti-N (COI)	Anti-S (U/ml)	WT nAb (IC_{50})	Omicron nAb (IC_{50})	Anti-N (COI)	Anti-S (U/ml)
**Uninfected**												
N1	5	5	0.09	<0.4	687	5	0.08	>250	144	5	0.07	>250
N2	5	5	0.07	<0.4	176	5	0.07	>250	146	179	0.06	>250
N3	5	5	0.07	<0.4	318	14	0.07	>250	192	124	0.06	>250
N4	5	5	0.06	<0.4	192	20	0.06	>250	61	17	0.06	>250
N5	5	5	0.07	<0.4	54	5	0.07	>250	30	5	0.06	>250
N6	5	5	0.08	<0.4	103	5	0.07	>250	63	5	0.06	>250
N7	5	5	0.07	<0.4	751	5	0.07	>250	276	12	0.06	>250
N8	5	5	0.06	<0.4	5	5	0.49	67.3	5	5	0.18	40.8
N9	5	5	0.07	<0.4	345	5	0.07	>250	204	39	0.06	>250
N10	5	5	0.15	<0.4	2041	262	0.16	>250	191	17	0.13	>250
N11	5	5	0.09	<0.4	1108	5	0.09	>250	85	5	0.07	>250
N12	5	5	0.06	<0.4	211	5	0.07	>250	110	5	0.06	>250
N13	5	5	0.07	<0.4	147	5	0.07	>250	104	5	0.06	>250
N14	5	5	0.07	<0.4	511	5	0.07	>250	58	5	0.06	>250
N15	5	5	0.07	<0.4	5	5	0.07	>250	22	5	0.06	>250
N16	5	5	0.06	<0.4	781	5	0.07	>250	43	5	0.06	>250
N17	5	5	0.06	<0.4	392	5	0.07	>250	98	5	0.06	>250
N18	5	5	0.07	<0.4	5	5	0.08	>250	5	5	0.07	>250
N19	5	5	0.06	<0.4	312	31	0.06	>250	40	12	0.06	>250
N20	5	5	0.07	<0.4	137	5	0.08	>250	16	5	0.07	>250
N21	5	5	0.07	<0.4	198	5	0.08	>250	74	5	0.07	>250
N22	5	5	0.07	<0.4	134	13	0.07	>250	74	105	0.06	>250
N23	5	5	0.07	<0.4	1190	174	0.07	>250	189	5	0.06	>250

N24	5	5	0.07	<0.4	333	12	0.07	>250	79	5	0.06	>250
N25	5	5	0.06	<0.4	5	5	0.07	>250	12	12	0.06	>250
**Post COVID-19**												
P1	81	5	73.3	>250	4753	437	98.5	>250	830	188	71.2	>250
P2	5	5	46.0	103.0	27	5	6.9	>250	96	5	2.8	71.0
P3	5	5	19.3	45.8	2833	522	18.6	>250	298	142	8.8	>250
P4	5	5	86.7	>250	3086	4234	0.07	>250	915	2591	49.2	>250
P5	5	5	8.9	214.0	1076	318	0.07	>250	226	117	7.0	>250
P6	5	5	58.1	>250	1557	482	0.07	>250	452	209	15.5	>250
P7	720	78	108.0	>250	7360	2862	0.07	>250	4317	6777	138.0	>250
P8	13	5	152.0	>250	1336	5917	0.07	>250	526	3175	61.5	>250

*nAb refers to neutralizing antibody
