# Supplementary material for: Humoral and T Cell Immune Responses against SARS-CoV-2 after Primary and Homologous or Heterologous Booster Vaccinations and Breakthrough Infection: A Longitudinal Cohort Study in Malaysia
Source: Viruses. 2023 Mar 25;15(4):844. doi: 10.3390/v15040844 (PMC10146761; doi:10.3390/v15040844)
Supplement: Supplementary file 1 [file viruses-15-00844-s001.zip › Table S3.pdf]

Table S3. Pseudovirus neutralization assay against SARS-CoV-2 wild type and Omicron spike, Elecsys Anti-SARS-CoV-2 S and Elecsys Anti-SARS-CoV-2 results for homologous booster, heterologous booster, and breakthrough infection groups at time-points before the booster dose (B1), and at 21 days (B2), 3 months (B3) and 6 months after the booster dose (B4).

Group	B1				B2				B3				B4			
	WT nAb (IC_{50})	Omicron nAb (IC_{50})	Anti-N (COI)	Anti-S (U/ml)	WT nAb (IC_{50})	Omicron nAb (IC_{50})	Anti-N (COI)	Anti-S (U/ml)	WT nAb (IC_{50})	Omicron nAb (IC_{50})	Anti-N (COI)	Anti-S (U/ml)	WT nAb (IC_{50})	Omicron nAb (IC_{50})	Anti-N (COI)	Anti-S (U/ml)
**Homologous booster**																
A1	5	5	0.06	>250	551	167	0.07	>250	138	5	0.06	>250	928	5	0.07	>250
A2	179	5	0.06	>250	2081	408	0.06	>250	668	470	0.07	>250	1100	332	0.06	>250
A3	31	5	0.13	>250	6751	947	0.07	>250	215	239	0.13	>250	1115	404	0.14	>250
A4	5	5	0.06	183.0	1519	61	0.06	>250	156	31	0.07	>250	638	5	0.07	>250
A5	311	5	0.06	>250	5042	773	0.06	>250	4073	680	0.06	>250	2212	116	0.07	>250
A6	76	5	0.07	210.0	3359	295	0.07	>250	977	212	0.07	>250	474	207	0.07	>250
A7	5	5	0.30	>250	856	218	0.30	>250	533	5	0.30	>250	97	13	0.26	>250
A8	529	5	0.07	>250	1381	598	0.07	>250	1844	422	0.07	>250	1480	672	0.08	>250
A9	5	5	0.06	>250	638	158	0.07	>250	438	5	0.06	>250	21	5	0.06	>250
A10	42	5	0.06	>250	1095	270	0.06	>250	1703	164	0.07	>250	747	148	0.07	>250
A11	41	5	0.17	>250	1234	449	0.17	>250	518	124	0.17	>250	64	19	0.17	>250
A12	86	11	0.06	>250	1539	833	0.06	>250	1558	684	0.06	>250	1280	638	0.06	>250
A13	111	19	0.06	>250	11436	1925	0.06	>250	2435	2283	0.06	>250	4507	1005	0.06	>250
A14	36	5	0.06	>250	784	101	0.06	>250	304	92	0.06	>250	83	27	0.07	>250
**Heterologous booster**																
B1	5	5	0.06	199.0	766	389	0.06	>250	179	321	0.06	>250				
B2	5	5	0.07	140.0	914	76	0.07	>250	265	79	0.07	>250				
B3	5	5	0.06	150.0	1329	269	0.06	>250	301	178	0.06	>250				
B4	5	5	0.06	>250	1044	463	0.06	>250	722	298	0.07	>250				
B5	5	5	0.06	>250	1789	55	0.07	>250	613	5	0.07	>250				
B6	413	5	0.06	>250	1722	187	0.07	>250	222	107	0.06	>250				
B7	5	5	0.06	>250	1582	98	0.06	>250	127	191	0.06	>250				
B8	5	5	0.08	>250	1865	255	0.09	>250	507	202	0.09	>250				
B9	5	5	0.06	>250	1442	392	0.07	>250	713	700	0.07	>250				
B10	5	5	0.09	37.5	338	129	0.09	>250	442	250	0.09	>250				
B11	5	5	0.06	220.0	1186	57	0.06	>250	67	27	0.07	>250				

B12	5	5	0.06	126.0	412	98	0.07	>250	45	373	0.07	>250				
B13	5	5	0.06	223.0	2462	275	0.06	>250	284	207	0.06	>250				
B14	5	5	0.06	226.0	1661	87	0.06	>250	82	24	0.07	>250				
B15	5	5	0.07	96.3	480	169	0.08	>250	303	72	0.07	>250				
**Homologous booster breakthrough infection**																
C1	47	5	0.06	>250	760	272	0.06	>250	240	184	0.06	>250	1948	1346	4.04	>250
C2	5	5	0.07	143.0	1252	28	0.07	>250	93	5	0.07	>250	1107	1505	29.00	>250
C3	114	5	0.06	>250	5711	1129	0.06	>250	5363	503	0.06	>250	7423	5528	21.10	>250
C4	5	5	0.06	98.6	11824	853	0.07	>250	95	5	0.07	>250	305	260	30.20	>250
C5	75	5	0.06	>250	4446	238	0.06	>250	788	161	0.06	>250	5496	6005	8.90	>250
C6	45	5	0.07	>250	2274	465	0.06	>250	907	229	0.07	>250	3878	2288	9.00	>250
C7	77	5	0.07	>250	1789	443	0.06	>250	899	176	0.06	>250	3214	1037	13.60	>250
C8	5	5	0.11	>250	803	368	0.11	>250	315	190	0.10	>250	2077	4190	19.60	>250
C9	82	5	0.09	>250	916	628	0.09	>250	1532	856	0.09	>250	6804	3713	154.00	>250
C10	5	5	0.06	158.0	1182	786	0.06	>250	1063	437	0.06	>250	5690	5082	40.20	>250
C11	5	5	0.07	>250	1115	208	0.07	>250	1113	260	0.07	>250	2909	997	20.30	>250
