# Supplementary material for: Humoral and T Cell Immune Responses against SARS-CoV-2 after Primary and Homologous or Heterologous Booster Vaccinations and Breakthrough Infection: A Longitudinal Cohort Study in Malaysia
Source: Viruses. 2023 Mar 25;15(4):844. doi: 10.3390/v15040844 (PMC10146761; doi:10.3390/v15040844)
Supplement: Supplementary file 1 [file viruses-15-00844-s001.zip › Table S4.pdf]

Table S4. Statistical comparison of T cell responses against Ag1, Ag2 and Ag3 in homologous booster, heterologous booster, and breakthrough infection groups at time-points before the booster dose (B1), and 21 days (B2), 3 months (B3) and 6 months after the booster dose (B4).

Group	Time-points			
	B1	B2	B3	B4
**Homologous booster**				
Ag1 vs Ag2	ns	0.0017	0.0269	0.0025
Ag2 vs Ag3	0.036	ns	0.001	0.0067
Ag1 vs Ag3	0.0001	0.0001	0.001	0.0021
**Heterologous booster**				
Ag1 vs Ag2	ns	ns	0.019	
Ag2 vs Ag3	ns	0.0034	ns	
Ag1 vs Ag3	0.0144	0.0017	0.0137	
**Homologous booster breakthrough infection**				
Ag1 vs Ag2	ns	0.0137	ns	0.0137
Ag2 vs Ag3	ns	ns	ns	ns
Ag1 vs Ag3	ns	0.0186	ns	0.0098
